# Functional roles of DExD/H-box RNA helicases in Pre-mRNA splicing

**DOI:** 10.1186/s12929-015-0161-z

**Published:** 2015-07-16

**Authors:** Yen-Chi Liu, Soo-Chen Cheng

**Affiliations:** Institute of Molecular Biology, Academia Sinica, Taipei, Taiwan 115 Republic of China

**Keywords:** DExD/H-box RNA helicase, Splicing, Spliceosome, RNA processing

## Abstract

Splicing of precursor mRNA takes place *via* two consecutive steps of transesterification catalyzed by a large ribonucleoprotein complex called the spliceosome. The spliceosome is assembled through ordered binding to the pre-mRNA of five small nuclear RNAs and numerous protein factors, and is disassembled after completion of the reaction to recycle all components. Throughout the splicing cycle, the spliceosome changes its structure, rearranging RNA-RNA, RNA-protein and protein-protein interactions, for positioning and repositioning of splice sites. DExD/H-box RNA helicases play important roles in mediating structural changes of the spliceosome by unwinding of RNA duplexes or disrupting RNA-protein interactions. DExD/H-box proteins are also implicated in the fidelity control of the splicing process at various steps. This review summarizes the functional roles of DExD/H-box proteins in pre-mRNA splicing according to studies conducted mostly in yeast and will discuss the concept of the complicated splicing reaction based on recent findings.

## Introduction

RNA splicing is a fundamental process in eukaryotic gene expression and is also highly regulated in higher eukaryotic cells. Many human diseases are associated with splicing defects or are caused by splicing misregulation [1, 2]. The splicing reaction requires five small nuclear RNAs (snRNAs), U1, U2, U4, U5 and U6, and a large number of proteins [3–5]. These factors assemble on the pre-mRNA to form a large ribonucleoprotein complex, called the spliceosome, on which the catalytic reactions take place.

The splicing cycle can be divided into four phases: spliceosome assembly, spliceosome activation, catalytic reactions, and spliceosome disassembly. Spliceosome assembly involves sequential binding of the five snRNAs, in the form of small nuclear ribonucleoprotein particles (snRNPs), to the pre-mRNA. The snRNAs play roles in the recognition and alignment of splice sites. Upon the binding of all five snRNAs, the spliceosome undergoes a major structural rearrangement, releasing U1 and U4, to form the active spliceosome, which can then catalyze two steps of the transesterification reaction, generating lariat intermediates and products. After the reaction, the spliceosome first releases the mature message and then is disassembled so that the splicing factors can be recycled [6, 7].

The structures of the catalytic core of the spliceosome and self-splicing group II introns are highly similar. They also share the same chemical mechanism and are believed to be evolutionarily related. For these reasons, pre-mRNA splicing is thought to also be an RNA-based reaction, with protein factors to support and modulate the structure of RNA in the catalytic core. Accumulating evidence supports RNA-catalyzed splicing of spliceosomal introns [8–12]. The structures of group II introns have recently been solved [13–15]. Several structures from different catalytic states have been determined and have provided structural and mechanistic insights into how the introns switch conformations between steps to position the splice sites [16].

Structural changes of the spliceosome are mediated by DExD/H-box RNA helicases [17–19]. DExD/H-box proteins are a family of RNA-dependent ATPases (or NTPases), which utilize the energy from ATP hydrolysis to modulate the structure of RNA or ribonucleoprotein complexes [20, 21]. Eight DExD/H-box proteins are required for the splicing reaction, two for each phase of the spliceosome pathway [22, 23]. Prp5 and Sub2 are required for the assembly phase of the spliceosome, with both involved in the formation of the prespliceosome [24–26]. Prp28 and Brr2 are required for activation of the spliceosome in releasing of U1 and U4, respectively [27–29]. Prp2 and Prp16 are required for each of the catalytic steps, and Prp22 and Prp43 are required for disassembly of the spliceosome [30–35]. Besides their ATPase-associated functions, Prp2, Prp5, Prp16 and Prp22 have been shown to have an ATP-independent function in the splicing pathway [25, 36–38]. Prp5, Prp16, Prp22 and Prp28 have also been demonstrated to play roles in the control of splicing fidelity [37, 39–44]. In this review, we summarize the functional roles of these proteins in the spliceosome pathway and discuss the underlying mechanisms of their functions.

## Review

### Overview of pre-mRNA splicing pathway

The spliceosome is assembled in a stepwise manner through ordered binding of the five snRNAs and protein factors to the pre-mRNA [6, 7]. U1 recognizes the 5′ splice site through RNA base pairing with the 5′ splice site sequence, and binds to the pre-mRNA to form the commitment complex (CC), which can be resolved into two complexes, CC1 and CC2, by gel electrophoresis. Formation of the commitment complex does not require ATP [45]. U2 binds to the branch site sequence, also through RNA base pairing, to form the prespliceosome. Although U1 normally binds to the pre-mRNA prior to U2 binding, U2 can bind to the branch site independent of U1 binding in mammalian extracts [46]. After binding of U1 and U2 snRNPs, U4, U5 and U6 are recruited to the spliceosome as a pre-formed U4/U6.U5 tri-snRNP. Neither U4/U6 di-snRNP or U5 snRNP alone binds to the spliceosome [47].

Loading of the tri-snRNP marks the end of the assembly phase of the spliceosome. The spliceosome then undergoes a dramatic structural rearrangement, releasing U1 and U4, and forming new base pairs between U2 and U6 and between U6 and the 5′ splice site [6, 7]. RNA-RNA base pairings form the framework of the catalytic core of the spliceosome, which is highly similar to that of self-splicing group II introns [48, 49]. The structure is presumably stabilized and modulated by protein factors. A protein complex associated with Prp19, named NTC for *N*ine*T*een *C*omplex[50], is required for stable association of U5 and U6 with the spliceosome after the release of U1 and U4 to direct specific interactions of U5 and U6 with pre-mRNA [50–52]. Other proteins may be also involved in fine-tuning the RNA structure during the splicing reaction.

The catalytic reaction comprises two consecutive steps of transesterification. Each of the catalytic steps involves an ATP-dependent step, which requires a DExD/H-box protein, and an ATP-independent step, which requires a specific set of proteins to promote the catalytic reaction. Exchange of proteins modulates structural changes of the spliceosome during the catalytic reaction. After completion of the reaction, the spliceosome first releases the mRNA and is then disassembled to recycle all components. Both steps require ATP and DExD/H-box proteins [6, 7, 53].

### DExD/H-box proteins in the splicing reaction

**Fig. 1:**
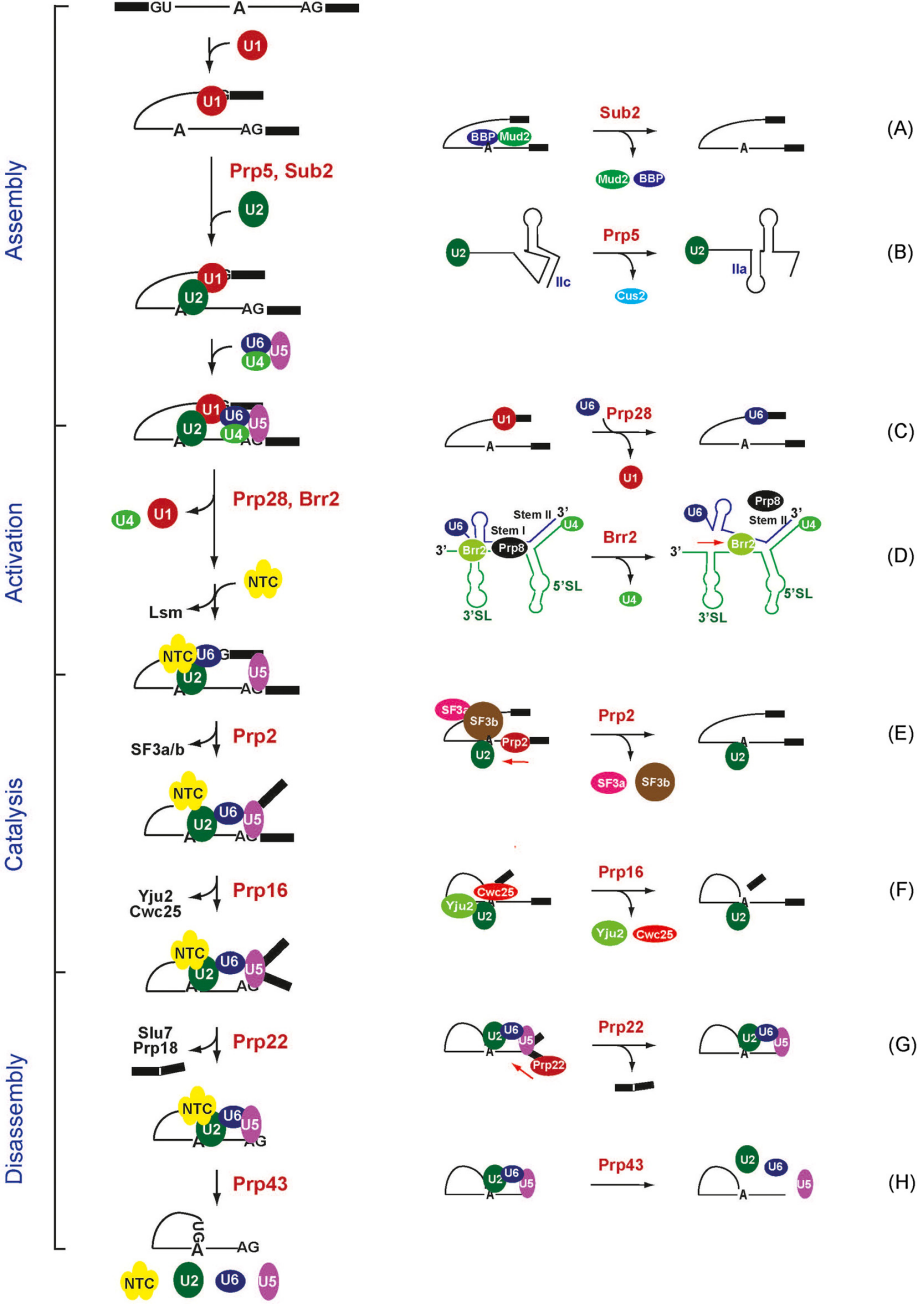
Schematic representation of the spliceosome pathway and the function of the DExD/H-box proteins involved. The spliceosome pathway can be divided into four phases: spliceosome assembly, spliceosome activation, catalytic steps and spliceosome disassembly. Two DExD/H-box proteins are required for each phase of the pathway, and their functions are shown. **a** Sub2 is implicated in the removal of BBP/Mud2 from binding to the branch site. **b** Prp5 is implicated in the removal of Cus2 from U2 snRNP and converts U2 snRNA into a functional form. **c** Prp28 is implicated in the removal of U1C or destabilization of U1 snRNA from the 5’ splice site to promote U1/U6 switch at the 5’ splice site. **d** Brr2 catalyzes U4/U6 unwinding to release U4. **e** Prp2 is required for remodeling of the spliceosome structure by destabilization of SF3a/b. Prp2 binds to the pre-mRNA in a region downstream of the branch site and translocates in a 3’-to’5’ direction to destabilize SF3a/b. **f** Prp16 is required for the release of Yju2/Cwc25. **g** Prp22 mediates the release mature mRNA, and **h** Prp43 mediates spliceosome disassembly. Prp22 binds to the mature mRNA in a region downstream of the splice junction and translocates in a 3’-to’5’ direction to destabilize mRNA from the spliceosome

Eight DExD/H-box proteins are involved in the splicing reaction, two for each phase of the pathway [22, 23]. In general they function as RNA chaperones to modulate the structure of RNA molecules or ribonucleoprotein complexes. Except for Brr2, which is an intrinsic component of U5 snRNP, all splicing DExD/H-box proteins only transiently interact with the spliceosome during the splicing reactions. Prp5, Sub2 and Prp28 belong to the DEAD-box family, and Brr2 belongs to the Ski2-like family. Both families of proteins exclusively use ATP for their functions. The four proteins involved in the catalytic step or disassembly, Prp2, Prp16, Prp22 and Prp43, belong to the DEAH/RHA family, and can use all four kinds of nucleotide triphosphates as energy sources [17–19]. These four proteins have been demonstrated to bind to the spliceosome in an ATP-independent manner, and are dissociated upon hydrolysis of ATP. They are retained on the spliceosome only when ATP is absent or the ATPase function of the protein is compromised. Mutations that abolish the ATPase activity frequently exhibit dominant-negative phenotypes. The functional roles of these proteins are illustrated in Fig. 1.

### Prp5 and Sub2 in spliceosome assembly

During early stage of spliceosome assembly, the branch binding protein BBP (branchpoint-binding protein; SF1 in human and Msl5 in yeast) binds to the branch site first and bridge the 5′ splice site and 3′ splice site by interacting with U1 snRNP proteins Prp40 and Mud2 (U2AF65 in human) [54–56], which bind pre-mRNA in the 3′ splice site region [54, 55, 57]. BBP and Mud2, forming a heterodimer, are thought to recruit Sub2 to or near the branch site region to mediate the release of Msl5 and Mud2 and to allow U2 snRNP binding to the branch site [58, 59]. Although Sub2 is essential for yeast under normal growth conditions, deletion of *MUD2* can bypass the requirement of *SUB2*, suggesting that an essential function of Sub2 is the removal of Mud2 to facilitate the association of U2 snRNP with the spliceosome [26]. It has also been shown that formation of CC2, but not CC1, requires Sub2 [60], indicative of an additional function of Sub2 in formation of the commitment complex. Besides functional roles in pre-mRNA splicing, Sub2 has also been reported to be involved in mRNA export [61–63].

Formation of the prespliceosome requires DExD/H-box protein Prp5. Prp5 has been shown to mediate the conformational change of U2 snRNP by hydrolysis of ATP, and its ATPase activity is stimulated to a much higher level by U2 snRNA than by other snRNAs or nonspecific RNAs [64]. Several U2 snRNP components, including Prp9, Prp11, Prp21, Cus1, Cus2 and stem IIa of U2 snRNA, genetically interact with Prp5 [65–68], thus functionally linking Prp5 and U2 snRNP. In the absence of Cus2, the formation of prespliceosome requires Prp5, but the ATPase function of Prp5 is dispensable for cellular growth and splicing [25]. Prp5 was thus proposed to promote the formation of stem IIa by displacing Cus2 from U2 in an ATP-dependent manner to form a functional U2 snRNP that associates with the spliceosome, and Prp5 has an additional ATP-independent function in prespliceosome formation [24, 25]. Recently, Prp5 was shown to bind directly to U2 snRNA on binding to the spliceosome [40]. Prp5 is retained on the spliceosome when pre-mRNA carries mutations in the branch site, and only upon its release can tri-snRNP be recruited to the spliceosome [40].

In the fission yeast *Schizosaccharomyces pombe* and in human, Prp5 was shown to associate with U1 snRNPs and U2 snRNPs *via* its N-terminal domain, bridging U1 and U2 snRNPs to form the prespliceosome [68]. Due to the lack of the corresponding N-terminal U1-interacting domain, the bridging function of U1 and U2 snRNPs by Prp5 is not found in *Saccharomyces cerevisiae* [68].

Prp5 was previously proposed to play a role in splicing fidelity control to proofread the branch site by competing with base pairing between U2 snRNA and the branch site sequence in an ATP-dependent manner [39, 40]. A recent report reveals an alternative mechanism for Prp5 functions in proofreading the branch site sequence by counteracting tri-snRNP binding independent of ATP [40]. More details are discussed in a later section.

### Prp28 and Brr2 in spliceosome activation

Two DExD/H-box proteins, Prp28 and Brr2, are involved in spliceosome activation. Prp28 was implicated in the displacement of U1 in promoting U1/U6 switch at the 5′ splice site [29]. The requirement of Prp28 can be bypassed by mutations in U1 snRNP components, U1C (Yhc1 in yeast), Prp42, cap-binding protein Cbp80, and Ynl187, some of which are known to stabilize U1-5′ splice site interactions. This suggests that Prp28 may destabilize U1-5′ splice site interactions by destabilization of proteins binding to U1 snRNA or to the 5′ splice site [69, 70].

The human Prp28 ortholog (hPrp28, also known as DDX23) has an extra N-terminal domain with RS repeats that are phosphorylated by SRPK2 (serine/arginine protein-specific kinase 2). hPrp28 has been shown to associate with the tri-snRNP, and phosphorylation of Prp28 plays a role in regulating the recruitment of the tri-snRNP to the spliceosome [71]. Prp28 has also been demonstrated to proofread the 5′ splice site during spliceosome assembly [42] (see below).

Brr2 is an intrinsic component of U5 snRNP and is associated with the spliceosome through the binding of tri-snRNP. Brr2 is required for spliceosome activation by mediating the release of U4 [27, 28]. Brr2 has been demonstrated to unwind RNA duplexes *in vitro* [27, 28], and unwinding of the U4/U6 duplex can also occur on the purified snRNP [27], implicating Brr2 in mediating U4/U6 unwinding during spliceosome activation [27, 28, 72, 73]. Brr2 has been shown to load onto the single stranded region of U4 located downstream of U4/U6 stem I, and then to translocate along U4 in the 3′-to-5′ direction to disrupt stem I on separating U4/U6 duplex [74, 75]. Prp8 plays a central role in regulating the function of Brr2 on the spliceosome. The Jab/MPN domain in the C-terminal region of Prp8 has been shown to stimulate the unwinding activity of Brr2 [73], but the very C-terminal tail of Prp8 inhibits the RNA-binding, ATPase and U4/U6 unwinding activities of Brr2 [76]. The RNase H domain of Prp8 can also bind the same region of U4 where Brr2 binds, thus preventing the binding of Brr2 and inhibiting U4/U6 unwinding [74].

Brr2 is unusual among DExD/H-box proteins in that it contains two helicase domains [77]. The N-terminal helicase domain functions in unwinding activity [72], but the C-terminal helicase domain is catalytically inactive. Besides its role in mediating U4/U6 unwinding during spliceosome activation, Brr2 has additional roles in regulating the function of other splicing factors. The C-terminal domain of Brr2 interacts with many spliceosomal components, serving as protein-protein interaction platform [78, 79]. These proteins include Prp2, Prp16 and Slu7, which are involved in catalytic steps, and Ntr2, which is required for spliceosome disassembly [78, 80, 81]. It is possible that Brr2 serves as the platform for the recruitment of these splicing factors to the spliceosome at various stages of post-activated spliceosomes. Consistent with this notion, Prp16 and Slu7 have been shown to compete with the binding of Ntr2 to the spliceosome to prevent premature disassembly of the spliceosome [82]. The C-terminal Sec63-2 domain of Brr2 has been shown to interfere with RNA binding of Prp16, thereby modulating the ATPase activity of Prp16 [83]. Brr2 has also been implicated in the mediation of spliceosome disassembly [84], but whether its ATPase activity is involved remains controversial [85].

### Prp2, Prp16 and Prp22 in the catalytic step

The first catalytic reaction requires DExD/H-box protein Prp2 to remodel the spliceosome [31, 36, 86]. For its function, Prp2 requires the cofactor Spp2, which was initially identified as a multi-copy suppressor of the temperature-sensitive *prp2-1* mutant and shown to interact with Prp2 through its G-patch domain [87, 88]. Like Prp2, Spp2 can associate with the spliceosome prior to the first catalytic reaction and is released along with Prp2 upon ATP hydrolysis [87]. Although Spp2 was previously shown to be required for the recruitment of Prp2 to the spliceosome, a recent study using a purified splicing system revealed that Spp2 is dispensable for Prp2 recruitment, but functions in coupling the ATPase activity of Prp2 to remodeling of the spliceosome into a catalytically active form [89].

The function of Prp2 has recently been demonstrated to associate with destabilization of U2 snRNP components SF3a/b complexes from the spliceosome [36, 86]. SF3b component SAP155 and its *Saccharomyces cerevisiae* ortholog Hsh155 have been shown to crosslink to intron sequences flanking the branch site, suggesting a role of SF3b in stabilizing U2-branch site interaction during spliceosome formation [90, 91]. Conceivably, destabilization of SF3a/b allows exposure of the branchpoint and also relieves the rigidity of the catalytic center of the spliceosome so that RNA elements can interact to initiate the catalytic reaction. The function of Prp2 in the first catalytic step also requires an eIF4G-like protein, Cwc22 [92]. Cwc22 is not required for the recruitment of Prp2 to the spliceosome, but in its absence, Prp2 is dissociated from the spliceosome upon ATP hydrolysis without productive action [92].

Studies from proteomic and dual-color fluorescence cross-correlation spectroscopic (dcFCCS) analysis revealed that several other proteins, including Cwc24, Cwc27 and Bud13, are displaced during Prp2-mediated remodeling of the spliceosome, but the functions of these proteins are not known [93]. After the action of Prp2, high-affinity binding sites are created for Yju2 and Cwc25, which stabilize first-step conformation of the catalytic center of the spliceosome to promote the first reaction [93]. Prp2 has also been implicated in mediating an ATP-independent conformational change of the spliceosome, but the mechanism is unknown [36].

Prp2 has been shown to interact with the C-terminal region of Brr2 [81] and with pre-mRNA by UV-crosslinking analysis [81, 94]. A region of the intron sequence 23 to 33 nucleotides downstream of the branchpoint was shown to be necessary and sufficient for the ATP-dependent function of Prp2. It has been suggested that Prp2 is recruited to the spliceosome through interaction with Brr2 and then translocated to the pre-mRNA, which stimulates its ATPase activity. Hydrolysis of ATP powers Prp2 to move on the pre-mRNA in the 3′-to-5′ direction to destabilize SF3a/b [81].

The second catalytic reaction requires the DExD/H-box protein Prp16 and three other proteins, Slu7, Prp18 and Prp22 [38, 95, 96], although Prp22 was shown to be not required in a recent study using a purified splicing system [97]. Prp16 was originally identified as a suppressor of branchpoint A-to-C (or brC) mutant of the actin intron [98, 99], but was found to be only required for the second catalytic reaction *in vitro* [32]. Prp16 was proposed to induce a conformational change in the spliceosome prior to the second catalytic reaction, judging from the protection of the 3′ splice site from RNase H cleavage upon Prp16 action [100]. Although UV-crosslinking analysis revealed protection of the 3′ splice site may be due to Prp22 binding [101], other changes in the RNA structure of the spliceosome have also been observed. Genetic data have suggested that the U2 helix II switches from IIa to IIc conformation, and U2/U6 helix I is destabilized during the transition from the first to second catalytic step [102–104]. Since Prp16 can catalyze unwinding of synthetic RNA duplexes *in vitro* [105], it was proposed that Prp16 may mediate unwinding of the RNA duplex during the transition [102, 106]. However, it is also possible that change of the RNA structure is a consequence of protein displacement mediated by Prp16.

Recent studies have demonstrated dual roles of Prp16 in the catalytic step. After the first reaction, Prp16 is required for the displacement of Yju2 and Cwc25, which become tightly associated with the spliceosome [37]. This function in remodeling of the spliceosome requires ATP hydrolysis and results in stable association of Slu7/Prp18 to promote the second reaction [97]. As in the first step, the RNA structure in the catalytic center of the spliceosome is less rigid upon displacement of Yju2 and Cwc25. This allows positioning of the 3′ splice site to the catalytic center. *PRP16* has also been demonstrated genetic interactions with *ISY1*, *PRP8* and U6 snRNA, suggesting that they are potential targets of Prp16 [107–109]. Whether these factors interact with Prp16 directly or through interaction with Cwc25 remains unknown. Prp16 also has an ATP-independent function in the first catalytic step in promoting the binding of Cwc25 to impaired spliceosomes. Cwc25 binds tightly to the first-step spliceosome, but does not bind well to the pre-catalytic spliceosome. Although Prp16 normally binds to the spliceosome after the first reaction, under conditions that the reaction is impeded such as when pre-mRNA carries mutations at the branch site, Prp16 can bind to the spliceosome to stabilize the binding of Cwc25 to promote the first reaction without needing ATP [37].

### Prp22 and Prp43 in spliceosome disassembly

The primary function of Prp22 is to mediate the release of mature mRNA after completion of the splicing reaction [33]. Prp22 binds to the spliceosome together with Slu7 and Prp18 during the second catalytic step and was previously shown to be also required for the second reaction [38]. However, a recent study using a purified splicing system reported Prp22 is not required for the second reaction [93]. Prp22 binds directly to the intron sequence downstream of the branch site prior to the reaction [101] and translocates to the mRNA downstream of the exon-exon junction after exon ligation. It was proposed that Prp22 promotes disruption of mRNA/U5 contacts by moving along mRNA in the 3′-to-5′ direction in releasing the mRNA from the spliceosome [110]. Prp22 has been demonstrated to unwind RNA duplexes *in vitro* [38, 111], and the helicase activity is essential for mRNA release [112], suggesting that Prp22 might mediate unwinding of mRNA/U5 base pairings. On the other hand, genetic data showed that Prp8-Arg1753 mutants suppress Prp22 helicase-defective mutants [113] as well as specific U5 loop 1 mutant alleles [114]. These results suggest that Prp8-Arg1753 may play a role in stabilizing U5/exon interactions before exon ligation, and Prp22 may function in disrupting RNA-RNA or RNA-protein interactions that are normally stabilized by Prp8.

Prp43 is the key player in mediating spliceosome disassembly [34, 35, 115]. It mediates disassembly of the spliceosome normally after completion of the splicing reaction, but can also mediate disassembly of the impaired spliceosome intermediates or spliceosomes arrested in the middle of the pathway [82, 116]. The function of Prp43 requires two co-factors, Ntr1 (also called Spp382) and Ntr2, which form a dimeric complex for the recruitment of Prp43 to the spliceosome [35, 117, 118]. Prp43 can also associate with Ntr1-Ntr2 to form a functional NTR complex, which catalyzes disassembly of the affinity-purified spliceosome in the presence of ATP [35]. Ntr1 interacts with both Ntr2 and Prp43 on formation of the NTR complex. The G-patch domain of Ntr1 interacts with Prp43 and stimulates the helicase activity of Prp43 [119]. Ntr2 interacts with U5 component Brr2, and this interaction is proposed to mediate the recruitment of NTR to the spliceosome [80]. Prp43 has been demonstrated to unwind RNA duplexes *in vitro* [120], but whether Prp43 catalyzes RNA unwinding on the spliceosome to mediate disassembly is not clear. Another DExD/H-box protein Brr2 has also been implicated in the disassembly by disruption of U2/U6 base pairings [84]. However, a recent study argues against the involvement of the ATPase function of Brr2 since, while Brr2 is ATP-specific, all four nucleotide triphosphates could be used as the energy source in the disassembly assay using a purified splicing system [85].

Prp43 has been demonstrated to be functionally linked to discard of spliceosome intermediates rejected from the pathway [115]. Dissection of the pathway revealed that only specific intermediate complexes, those formed after the action of DExD/H-box proteins Prp2 and Prp16, are susceptible to Prp43-mediated disassembly, suggesting a function of DExD/H-box proteins in marking the spliceosome for susceptibility to the disassembly machinery, in accordance with their proposed roles in splicing fidelity control [82].

### DExD/H-box proteins in splicing fidelity control

The first insight into the involvement of DExD/H-box proteins in splicing fidelity control came from the isolation of *PRP16* mutants as suppressors to branchpoint A-to-C mutation [98, 99]. The level of suppression was found to inversely correlate with that of the ATPase activity of Prp16. A model for a role of Prp16 in splicing fidelity control by a kinetic proofreading mechanism was proposed [44, 121]. In this model, Prp16 can direct the impaired spliceosome to a discard pathway coupling the energy from ATP hydrolysis. A conformational change in the spliceosome, affected by the sequence in the branchpoint, competes with the action of Prp16 to prevent the spliceosome from being rejected. While mutation in the branchpoint impedes the conformational change of the spliceosome, it allows Prp16 to act on and reject the impaired spliceosome. Reducing the ATPase activity of Prp16 allows more time for the spliceosome to proceed through the conformational change needed for progression to the normal pathway. Based on genetic data, Prp2, Prp5, Prp22 and Prp28 have been proposed to play roles in proofreading the catalytic core of the spliceosome, the branch site, 3′ splice site and the 5′ splice, respectively, by similar mechanisms [37, 39–44, 122]. Recent biochemical analysis has revealed an ATP-independent function of Prp16 in promoting the first catalytic reaction by stabilizing the binding of Cwc25 [37]. While splicing of branchpoint-mutated pre-mRNA is completely blocked in the absence of Prp16, ATPase-deficient Prp16 allows progression of the first reaction to a large extent. Based on this finding, it was proposed that Prp16-mediated rejection of impaired spliceosomes might be a consequence of Cwc25 displacement prior to the catalytic reaction, rendering the spliceosome susceptible to NTR for disassembly. The relative ratio of the ATP-independent to ATP-dependent function of Prp16 determines the efficiency of splicing proceeding through the first reaction [37, 123]. In this regard, Prp16 may only play a passive role in proofreading the branch site sequence. This also raises a question of whether all splicing DExD/H-box proteins share a common feature of controlling splicing fidelity by the ATP-dependent proofreading mechanism. At least for Prp5, an alternative ATP-independent mechanism explaining the suppression effect of branch site mutants by Prp5 was recently proposed [40].

## Conclusions

Originating from self-splicing group II introns, the spliceosome has evolved into a highly sophisticated structure that requires eight DExD/H-box proteins to mediate its structural rearrangements along the splicing pathway. These proteins play roles in remodeling of the spliceosome by either unwinding of RNA duplexes or disrupting RNA-protein interactions to facilitate structural changes of the spliceosome. Under conditions that structural changes can be more easily achieved, the function of DExD/H-box proteins can be dispensable. Although several DExD/H-box proteins have been demonstrated to play roles in proofreading splice sites based on genetic data, studies using biochemical approaches have shed more insights into the functional roles of these proteins, and provided new explanations for previous genetic data by alternative mechanisms. It is necessary to analyze the function of these proteins in more details to elucidate the mechanism of splicing fidelity control.
